# Adaptation to implied tilt: extensive spatial extrapolation of orientation gradients

**DOI:** 10.3389/fpsyg.2013.00438

**Published:** 2013-07-19

**Authors:** Neil W. Roach, Ben S. Webb

**Affiliations:** Visual Neuroscience Group, School of Psychology, The University of NottinghamNottingham, UK

**Keywords:** adaptation, psychological, tilt aftereffect, texture analysis, orientation, cortical plasticity

## Abstract

To extract the global structure of an image, the visual system must integrate local orientation estimates across space. Progress is being made toward understanding this integration process, but very little is known about whether the presence of structure exerts a reciprocal influence on local orientation coding. We have previously shown that adaptation to patterns containing circular or radial structure induces tilt-aftereffects (TAEs), even in locations where the adapting pattern was occluded. These spatially “remote” TAEs have novel tuning properties and behave in a manner consistent with adaptation to the local orientation implied by the circular structure (but not physically present) at a given test location. Here, by manipulating the spatial distribution of local elements in noisy circular textures, we demonstrate that remote TAEs are driven by the extrapolation of orientation structure over remarkably large regions of visual space (more than 20°). We further show that these effects are not specific to adapting stimuli with polar orientation structure, but require a gradient of orientation change across space. Our results suggest that mechanisms of visual adaptation exploit orientation gradients to predict the local pattern content of unfilled regions of space.

## Introduction

Analysis of orientation structure is fundamental to many aspects of visual perception, including the ability to parse the retinal image into distinct regions and identify the form of different objects. To achieve these goals, the visual system must first encode local orientation signals at different points in the visual field before integrating this information across space. Representation of local orientation is typically associated with primary visual cortex (V1), which is characterized by an orderly mapping of receptive field location and orientation preference across the cortical surface (Hubel and Wiesel, 1968; Blasdel and Salama, 1986; Wandell et al., 2007). Interaction between neighboring neurons provides a potential means to begin extracting orientation structure beyond the spatial constraints of an individual receptive field. For example, long-range excitatory horizontal connections in V1 linking regions of similar orientation preference (Gilbert and Wiesel, 1979, 1983, 1989) have been proposed as a mechanism for integrating along contours (Kapadia et al., 1995, 2000; Li et al., 2006). However, it is likely that more complex and spatially extensive orientation structure analysis relies upon the progressive convergence of V1 outputs in extra-striate visual areas.

Progress is being made toward understanding the types of structure represented at intermediate levels of the processing hierarchy. While the majority of neurons in V2 display spatially homogenous orientation tuning comparable to that seen in V1, sub-populations have been identified that exhibit distinct preferences for orientation discontinuities (Nishimoto et al., 2006; Anzai et al., 2007; Schmid et al., 2009) and texture boundaries (El-Shamayleh and Movshon, 2011). Selectivity to higher order shape properties such as contour curvature (Pasupathy and Connor, 1999, 2001) and polar form (Gallant et al., 1996) has been reported in V4, where neurons have larger receptive fields and begin to show sensitivity to the relative (rather than absolute) positioning of orientations within their receptive fields. These neurophysiological findings are complemented by a growing body of psychophysical studies examining the spatial integration of orientation signals in tasks such as texture segregation (Nothdurft, 1985; Landy and Bergen, 1991), contour detection (Field et al., 1993; Hess et al., 2003), symmetry detection (Dakin and Herbert, 1998; Wilson and Wilkinson, 2002), structure detection (Wilson et al., 1997; Wilson and Wilkinson, 1998; Dakin, 1999; Webb et al., 2008), shape discrimination (Wilson and Wilkinson, 1998; Wilkinson et al., 1998) and contrast detection (Meese and Summers, 2007; Meese et al., 2007; Meese, 2010). Together, this work is providing insight into the mechanisms underpinning human sensitivity to orientation structure of varying complexity and spatial scale.

While the majority of studies in this area tends to focus on the feed-forward pooling of local orientation signals to extract global structure, an important but relatively under-explored question is whether the presence of structure exerts a reciprocal influence on local orientation coding. We know that the responses of single neurons in V1 are strongly modulated by stimulation in the space surrounding the receptive field (e.g., Blakemore and Tobin, 1972; Kapadia et al., 1995, 2000; Webb et al., 2005), but evidence for any selectivity to global orientation structure across large regions of space is currently lacking (Smith et al., 2002). Functional imaging studies have demonstrated that BOLD responses in V1 are systematically suppressed when images contain coherent shapes or objects compared to when they have random orientation structure (Murray et al., 2002; Rainer et al., 2002; Murray, 2004). These findings are consistent with hierarchical predictive coding models, which posit that feedback from higher-level areas acts to remove or “explain away” the predictable components of signals, thereby reducing redundancy in the neural representation (Mumford, 1991; Rao and Ballard, 1999; Spratling, 2010). Predictive coding has been shown to provide an elegant account of a variety of response properties in the retina (Srinivasan et al., 1982), lateral geniculate nucleus (Dong and Atick, 1995; Dan et al., 1996), and V1 (Rao and Ballard, 1999; Spratling, 2010). However, the precise nature of the interaction between local orientation coding and higher-order structure processing remains unclear. For example, in some instances BOLD responses in V1 appear to increase with the presence of orientation structure rather than decrease (Altmann et al., 2003; Kourtzi et al., 2003). It has also been argued that local orientation variability may be the prime determinant of the observed changes in V1 response, rather than the degree of coherent structure *per se* (Dumoulin, 2006).

In a previous psychophysical study, we investigated the impact of global orientation structure on the adaptation of local orientation coding mechanisms (Roach et al., 2008). Following passive exposure to a large, circular grating centered on fixation, observers discriminated the orientation of a small near-vertical Gabor test stimulus presented at different locations along an iso-eccentric ring. An annular region of the circular stimulus was occluded during adaptation, ensuring no spatial overlap occurred between the adapting and test patterns. Robust tilt aftereffects (TAEs) were observed in this occluded region, the direction and magnitude of which were consistent with adaptation to the orientation implied by the circular structure (but not physically present) at each test location. Earlier experiments investigating adaptation to partly-occluded visual patterns have been criticized on the basis that after-effects reported in occluded regions of space might be explainable in terms of a spatial spreading of local orientation adaptation effects from adjacent areas (see Sekuler et al., 1970; Weisstein, 1970). The properties of our spatially “remote” TAEs however, strongly suggest that they cannot be explained in this manner. Unlike traditional TAEs obtained following local adaptation (e.g., Ware and Mitchell, 1974), we found that remote TAEs were immune to manipulations of the relative spatial frequency of adapting and test patterns across several octaves. This produced an interesting double dissociation: whereas traditional TAEs obtained with matched adapt/test frequencies were on average ~2.5 times larger than the equivalent remote TAEs, this pattern was reversed when a three octave difference in spatial frequency was introduced. Spatially remote TAEs were also found to be selective to particular types of global orientation structure. Remote TAEs of comparable magnitude were obtained when observers adapted to radial, rather than circular patterns. However, little or no effect was found using simple iso-oriented grating adaptors with equivalent dimensions (Roach et al., 2008), making it unlikely that it is driven by local grouping processes (e.g., Sugita, 1999).

These remote TAEs are interesting for several reasons. Although a number of studies have suggested that high-level aftereffects may be inherited from adaptation occurring at early stages of visual processing (Xu et al., 2008, 2012; Dickinson et al., 2010), to our knowledge it is the only evidence suggesting that adaptation of a low-level stimulus property may be induced via feedback from processing at subsequent stages of analysis. In addition, because remote TAEs can be induced by adaptation to some types of orientation structure but not others, investigation of the factors driving this selectivity provides a novel opportunity to gain insight into the integration process itself. In the present study we extend our examination of these effects using texture patterns enabling us to flexibly manipulate the orientation structure present during adaptation.

## General methods

### Observers

Six observers participated in the study, the authors and four individuals who had previous experience of psychophysical observing, but were naive to the specific purposes of the study. All had normal or corrected-to-normal visual acuity.

### Stimuli

Stimuli were generated in Matlab and displayed via a Cambridge Research Systems ViSaGe system on a photometrically calibrated 22-inch Mitsubishi Diamond Pro 2045U CRT monitor. The display resolution was 1024 × 768 pixels, and at the viewing distance of 33.5 cm each pixel subtended a visual angle of 4 arcmin. The frame-rate was 100 Hz and the mean luminance of the display was 39 cd/m^2^.

**Figure 1:**
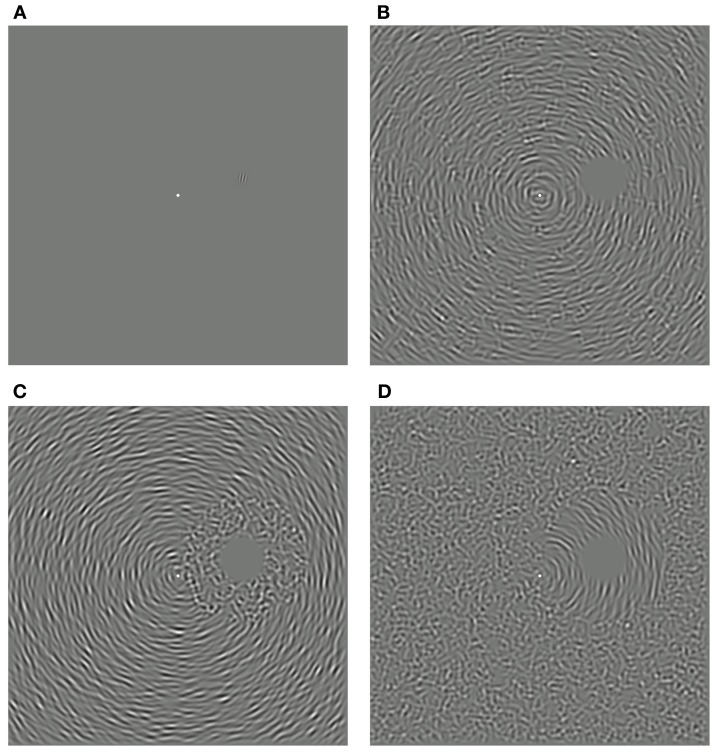
**Measuring the spatial specificity of the remote TAE**. **(A)** Example of a test stimulus, oriented clockwise of vertical and positioned above and to the right of the fixation dot. **(B)** Noisy concentric adapting stimulus comprising signal and noise elements randomly distributed throughout the texture pattern (“intermixed” condition). **(C)** Concentric adapting stimulus with all noise elements restricted to the space surrounding the test location (“proximal noise” condition). **(D)** Concentric adapting stimulus with all signal elements surrounding the test location (“distal noise” condition).

As depicted in Figure 1A, each test stimulus was a Gabor-like patch comprising a 2 c/° sine wave multiplied by a 1.33° diameter isotropic Hanning window. Test stimuli were presented 9.66° to the right and 2.59° above fixation (a polar angle of π/12 and eccentricity of 10°). The peak Michelson contrast of the test stimuli was 0.25.

Adapting stimuli were sequences of dense texture patterns, each formed by combining 5000 local oriented elements. Each oriented texture element was constructed in an identical manner to the target stimuli, but had a spatial frequency of 1 c/°. Different spatial frequencies were chosen for adapting and test stimuli to try and minimize the contribution of any local adaptation effects (see Roach et al., 2008). Texture elements were assigned a random phase and were centered at a random position within a 48 × 48° square region. Each adapting texture was normalized to ensure the mean luminance remained equal to the background and each had a RMS contrast of 9%. To minimize spatial overlap between the adapting and test stimuli, the contrast of the textures within a 3° radius circular region centered on the test location was set to zero. Beyond this region, contrast was restored gradually via a quarter-cycle cosine ramp over 1.6°. In each experiment, the orientation of each local element was determined by its location within the texture field and the desired orientation structure.

### Procedure

Participants were positioned in a chin rest and viewed the stimulus display binocularly in a darkened room. During a testing block, fixation was maintained on a small dot positioned in the center of the screen. Each block began with an initial 30 s period of adaptation, during which the adapting texture was regenerated every 100 ms to avoid the build-up of a retinal afterimage. Following a 500 ms blank inter-stimulus interval, a test stimulus was presented for 100 ms and the participant indicated whether it appeared to be tilted clockwise or counter-clockwise with respect to vertical. A further 3 s of top-up adaptation to the dynamic adapting texture preceded each subsequent trial. The orientation of the test stimulus was manipulated according to a method of constant stimuli, with 10 presentations of 7 linearly spaced orientations randomly ordered within a testing block. Participants completed 2–4 blocks per adaptation condition and breaks were taken between blocks to allow recovery from adaptation and avoid contamination across conditions. Psychometric functions were constructed for each condition and fitted with a logistic function using a maximum likelihood criterion, allowing estimation of the point of subjective equality (PSE): the physical orientation producing equal proportions of clockwise and counter-clockwise responses. The standard error associated with each PSE estimate was obtained via bootstrapping (Efron and Tibshirani, 1993). TAEs were inferred from the change in PSE relative to a baseline condition with no adaptation, with a positive change indicating a repulsive shift in the perceived orientation of the test stimulus away from the local orientation implied by the adapting structure.

## Experiment 1

### Methods

In our previous study, we induced remote TAEs at spatial locations coinciding with an occluded (i.e., zero-contrast) annular region of a circular adapting stimulus (Roach et al., 2008). To investigate the spatial extent over which this effect holds, we could have manipulated the size of this occluded region. However, this approach would have also altered the overall area and contrast energy of the adapter—a confound that we wished to avoid. Here we took a different approach using adapting textures composed of a variable proportion of signal and noise texture elements. Signal elements were assigned orientations consistent with a circular structure centered on fixation, whereas noise elements were assigned random orientations independent of their position. At the fixed test stimulus location, the tangential orientation implied by the circular structure was 15° counter-clockwise of vertical. This arrangement was chosen as it has been previously shown to produce the largest remote TAE (Roach et al., 2008).

Three variants of the noisy circular adaptor were used to investigate the spatial specificity of the remote TAE. In the “intermixed” condition, signal and noise elements were distributed throughout the texture pattern, as depicted in Figure 1B. In the “proximal noise” condition, all of the noise elements were restricted to an annular region surrounding the test location (Figure 1C). Conversely, in the “distal noise” condition, all of the signal elements surrounded the test location (Figure 1D). In each of the two segregated conditions, the outer radius of the annulus was manipulated. This enabled independent control of the spatial distance of signal texture elements relative to the test site and the overall coherence of the circular orientation structure (defined as the ratio of signal to noise elements). The overall area and RMS contrast of the adapting textures remained constant in all conditions. Note that in some conditions the outer radius of the annulus extended beyond the limits of the square texture region. However, this did not affect coherence calculations, which were based on the relative frequencies of visible texture elements.

### Results

**Figure 2:**
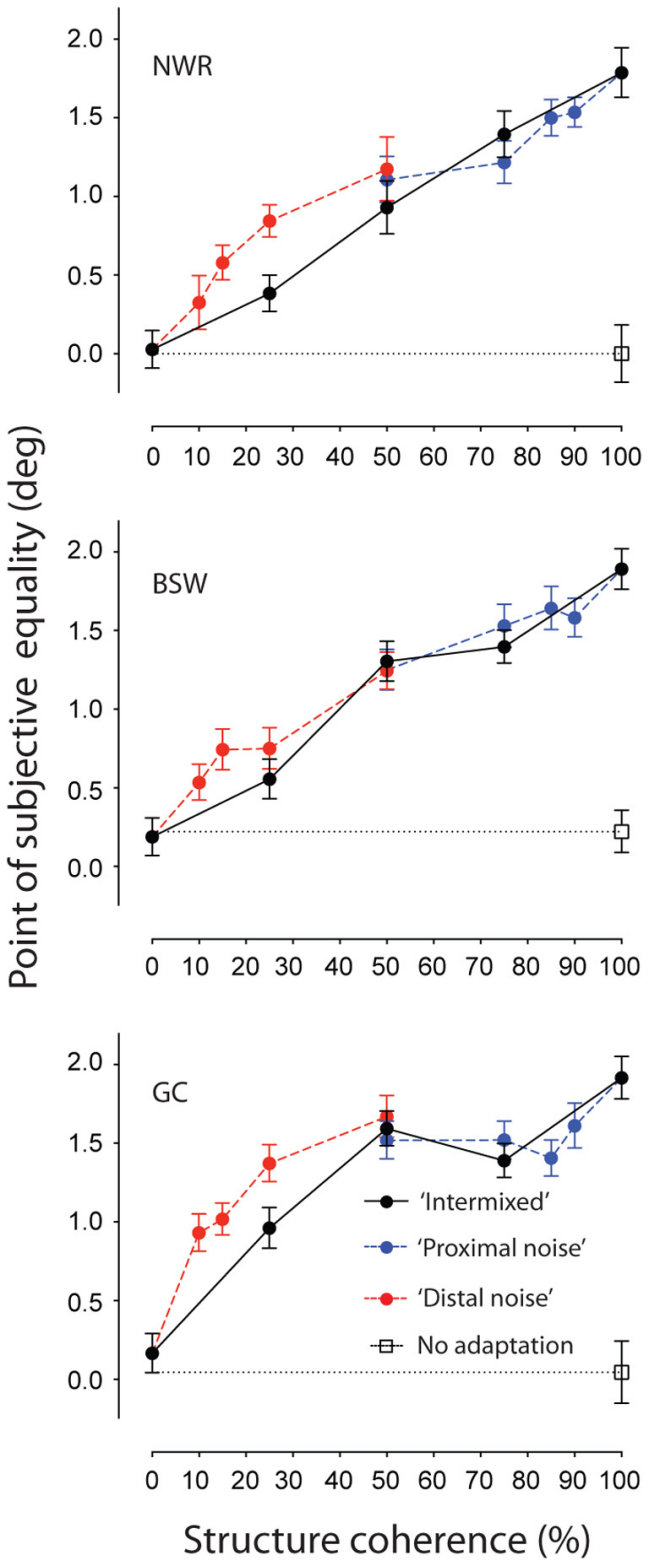
**Spatially remote TAEs-induced by adaptation to noisy concentric texture patterns with different spatial configurations**. Points of subjective equality are plotted as a function of signal to noise ratio for three individual observers. Black filled symbols represent performance where randomly oriented noise elements were distributed across the texture pattern (see Figure 1B). Red and blue symbols represent performance when noise elements were positioned inside (Figure 1C) or outside (Figure 1D) an annular region surrounding the test site, respectively. Unadapted (baseline) performance is shown by the unfilled black symbol. Error bars indicate ± 1 standard error.

Figure 2 shows individuals' PSEs plotted as a function of structure coherence for each of the different adapting configurations. When all of the local elements of the adapting texture had an orientation consistent with circular structure (i.e., 100% structure coherence) repulsive TAEs of approximately 2° were found for each observer. These effects are comparable in size to those previously reported with polar grating stimuli (Roach et al., 2008). Reducing the structure coherence of the adapting texture by introducing randomly oriented elements produced a concomitant reduction of the size of the TAE. Interestingly, this effect was largely robust to manipulations of the spatial configuration of signal and noise elements. Restricting the placement of noise elements to an annular region surrounding the test site (“proximal noise” condition, blue symbols) had no greater impact than randomly positioning them throughout the texture pattern (“intermixed” condition, black symbols). Instead, the two sets of data are virtually indistinguishable across the tested range of coherence values. This is remarkable because to achieve 50% structure coherence in the “proximal noise” condition, the required outer radius of the noise annulus was 22°, meaning that every texture element signaling the circular structure was at least this distance away from the center of the test site. Clearly, remote TAEs involve mechanisms operating across large regions of visual space.

Results for the complementary condition in which only signal elements were presented in the space surrounding the test location are indicated by the red symbols (“distal noise” condition). Again, TAE magnitude increased systematically as a function of coherence. However, shifts in PSE in this condition are larger than that observed with random positioning of signal and noise elements, suggesting there may be some contribution of local adaptation driven by regions of the adapting stimulus adjacent to the test region. To avoid this in subsequent experiments, we exclusively used adapting textures containing a proximal region of random orientation noise.

## Experiment 2

### Methods

Spatially remote TAEs can be induced by adaptation to circular or radial gratings, but not to simple Cartesian gratings (Roach et al., 2008). To better understand the reason for this discrepancy, we next investigated the contribution of two characteristics of polar gratings that do not apply to simple Cartesian gratings: the presence of orientation gradients and reflectional symmetry.

**Figure 3:**
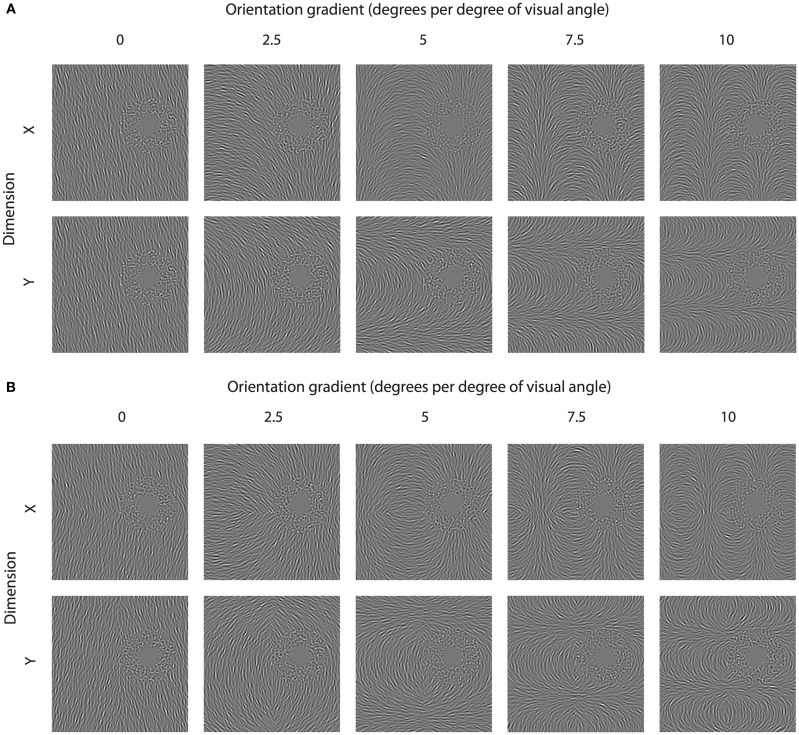
**Inducing spatially remote TAEs with orientation gradients**. **(A)** Smoothly varying orientation textures are shown, where structure is defined by a linear change in orientation as a function of either the horizontal (x) or vertical position (y). In each stimulus, the underlying orientation gradient is anchored about the test location (center of noisy annulus), ensuring that the implied orientation at that position is constant (15° counter-clockwise of vertical). **(B)** Orientation textures containing reflectional symmetry. Textures with linear orientation gradients along the x and y axes contain symmetry about the horizontal and vertical meridians, respectively.

Adapting textures were constructed in which the orientation of each element in the adapting texture was a linear function of either its horizontal or vertical position (see Figure 3A). The rate of change of orientation across space was manipulated between 0 (no change in orientation) and 10 degrees of rotation per degree of visual angle (see Figure 3A). In separate conditions, reflectional symmetry was introduced to adapting textures about the meridian of the axis along which the orientation gradient was applied. For example as shown in Figure 3B, textures with a change in orientation as a function of horizontal (x) position were made to be symmetrical about the horizontal midline. Note in Figure 3A, some conditions naturally contain reflectional symmetry about the axis orthogonal to the orientation gradients. In all cases, the orientation implied by the gradient at the test site remained constant at 15° counter-clockwise from vertical. To minimize the potential influence of local adaptation brought about by poor fixation stability, randomly oriented noise elements were presented in the region of space surrounding the test site (outer radius of annulus = 9.53°; structure coherence = 90%).

### Results

**Figure 4:**
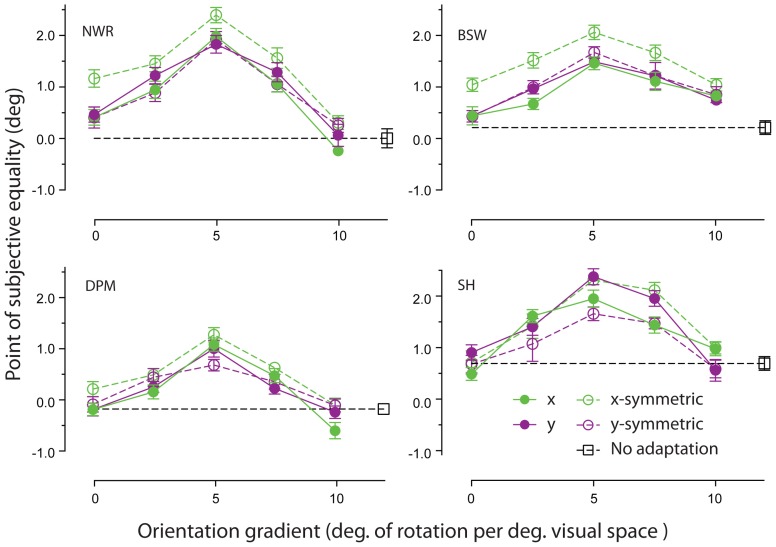
**Orientation gradient tuning of spatially remote TAEs**. PSEs for each observer are plotted as a function of the rate of change of orientation in the adapting texture across space, applied either in the horizontal (green symbols) or vertical (purple symbols) dimension. Unfilled and filled symbols indicate conditions with and without reflective symmetry applied around the horizontal or vertical meridian (see General Methods for details). Error bars indicate ± 1 standard error.

Adapting patterns containing a linear change in orientation across space were effective at inducing remote TAEs. As shown in Figure 4, the magnitude of observed effects displayed a tuned dependency on the gradient of the orientation change. For each observer, the largest shifts in PSE occurred for adapting textures in which orientation changed by 5° for each degree of visual angle. Remote TAEs in this gradient condition were comparable in size to those produced with high coherence circular structure in Experiment 1 (~2°). This similarity is noteworthy, because the orientation gradient of our circular textures was very similar to this peak value at the test location (5.73° change in orientation per unit space along arc at 10° eccentricity). Little or no consistent effect was observed across participants in the absence of a change in orientation across space, or where the orientation gradient approached 10 degrees of orientation change per degree of visual angle.

Comparison of the filled and unfilled symbols in Figure 4 suggests that remote TAEs are not sensitive to the degree of reflectional symmetry in the adapting stimulus. Although two participants displayed slightly larger remote TAEs when symmetry was applied around the horizontal meridian, in general no systematic pattern was observed across individuals.

## Experiment 3

### Methods

**Figure 5:**
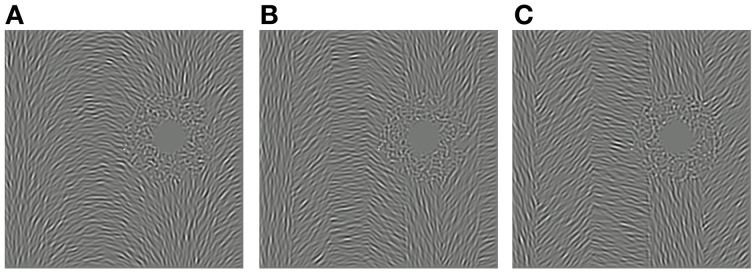
**Manipulating the smoothness of an orientation gradient via quantization of orientation within discrete spatial bands**. Each of the textures shown contain an constant linear change in orientation as a function of horizontal position, but have been quantized within bands measuring **(A)** 4° **(B)** 8°, or **(C)** 12°. Within each band all texture elements have a constant orientation, determined by space-averaging the underlying orientation gradient.

The importance of having a smooth change in orientation across space was investigated by quantizing the orientation gradient into discrete spatial regions along the gradient axis. A fixed orientation gradient (5° of rotation per degree of visual angle in the horizontal dimension) was used and the width of each spatial band was varied between 0.2 and 36° of visual angle (see Figure 5). Within a spatial band, the orientation of all local elements was set to the mean value of the underlying linear gradient. Spatial bands were positioned such that the center of the test region always coincided with the center of a band and was assigned an orientation of 15° counter-clockwise. Note that in the most extreme quantization condition tested (36°), the size of a band coincides with the spatial period of the orientation modulation. In this situation, averaging within a band completely removes the orientation gradient, resulting in an iso-oriented texture.

### Results

**Figure 6:**
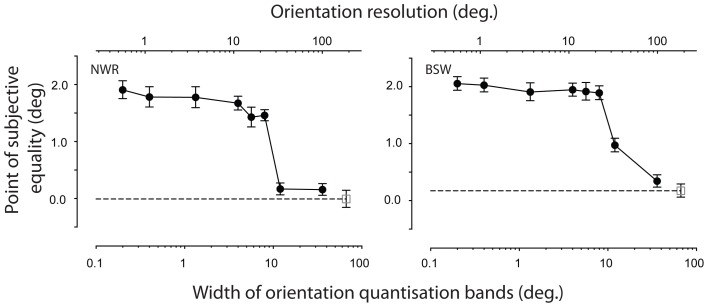
**Effect of quantizing the orientation gradient within the adapting texture**. PSEs are plotted as a function of the width of each spatial bin, within which all texture elements were assigned a fixed orientation (filled symbols). The upper scale shows the corresponding orientation resolution, defined as the change in orientation between successive spatial bands. For comparison, unfilled symbols indicate performance in the absence of adaptation. Error bars indicate ± 1 standard error.

The dependency of remote TAEs on the smoothness of the adapted orientation gradient is shown in Figure 6. Participants' results were insensitive to the introduction of small discontinuities in the orientation gradient, but the effect was abolished in the coarsest quantization conditions. Neither observer displayed a remote TAE when the width of each iso-oriented band exceeded ~12° of visual angle. This spatial band width corresponds to one third of the period of the underlying orientation gradient, meaning that each cycle of orientation change is signaled by three discrete orientations separated by 60° (see Figure 5C). The largest spatial band width at which consistent remote TAEs were observed was 8°. In this condition, the 40° change in orientation between each band is sufficient to produce a salient boundary percept (see Figure 5B), suggesting that perceptual segregation of the adapting texture into distinct regions is not the critical factor at play.

## Discussion

The visual system is often characterized as a hierarchy, in which successive stages analyse progressively more complex attributes of the visual scene. Adaptation is thought to occur at multiple stages, giving rise to a rich variety of perceptual aftereffects, ranging from distortions of basic stimulus properties such as local orientation (Gibson and Radner, 1937), to more complex higher-level structures (e.g., Suzuki, 2001; Peirce and Taylor, 2006; Anderson et al., 2007; Gheorghiu and Kingdom, 2007). However, surprisingly little is known about the interplay between changes occurring at multiple stages of analysis—a critical component of understanding adaptation in the visual system as a whole. In the present study we investigated the effect of adapting to images containing spatially-extensive orientation structure on the perceived orientation of small test stimuli. Replicating earlier findings (Roach et al., 2008), we were able to induce repulsive TAEs in regions of the visual field that did not receive input during adaptation. These remote TAEs cannot be explained by a spreading of local orientation adaptation effects across space, such as might result from fixational instability or some form of low-pass filtering by the visual system (i.e., optical or neural blur). Any mechanism of this sort would be highly dependent on the orientation content of the adapting stimulus within the region of space immediately surrounding the tested location. Counter to this prediction, the results of Experiment 1 indicate that placing a large annular field of random orientations around the test site does not prevent the induction of remote TAEs. Rather than being driven by local image content, these biases in perceived orientation are best explained in terms of extrapolation of the adapted orientation structure. Put simply, observers appear to adapt to the local orientation that is “implied” by the orientation structure of a nearby texture. Our results show that this extrapolation of adaptation effects to unfilled regions of the visual field is spatially extensive, spanning at least 22° of visual angle.

Previously we found that while adaptation to circular and radial patterns results in robust effects, adaptation to iso-oriented patterns does not. Several researchers have proposed that specialized mechanisms exist in the visual system for processing polar form (e.g., Wilson et al., 1997; Wilson and Wilkinson, 1998; Kurki, 2004; Dumoulin and Hess, 2007; Motoyoshi and Kingdom, 2010). However, the results of Experiment 2 show that remote TAEs are not specific to polar form *per se*, but do require some form of systematic change in orientation across space. The size of the effect is a tuned function of the adapting orientation gradient, peaking when orientation changes by approximately 5° for every degree of visual angle. This pattern of selectivity is an interesting result, as it runs counter to the large body of research showing that the visual system is especially sensitive to linking common orientations across space (for reviews see Hess et al., 2003; Loffler, 2008). In contrast to the tuning of the remote TAE, the ability of human observers to detect (Field et al., 1993; Geisler et al., 2001) and interpolate (Fulvio et al., 2008) contours typically decreases as a function of curvature. It is also established that extensive spatial summation occurs for iso-oriented textures both at and above threshold (Meese and Summers, 2007; Meese et al., 2007; Meese, 2010), so it is intriguing that adaptation to this form of orientation structure does not produce comparable remote aftereffects.

Our results raise the possibility that the visual system may have specialized mechanisms for processing orientation gradients contained in visual textures. Previous studies on orientation gradients have focused primarily on their role in texture segmentation. Perceptual segmentation tends to occur when the change in orientation between two regions (i.e., the orientation gradient across a border) is large relative to changes in orientation occurring within each region (e.g., Landy and Bergen, 1991; Nothdurft, 1992; Wolfson and Landy, 1995). It is possible that the mechanisms supporting texture segmentation may overlap with those underlying remote TAEs. However, the relationship between these phenomena is not clear. Several aspects of our results indicate that the generation of a remote TAE does not depend upon the adapting texture being perceived as a coherent surface. For example, the “proximal noise” and “distal noise” manipulations in Experiment 1 produce clear segmentation of the region surrounding the test site from the remainder of the adapting pattern (see Figure 1). Yet these stimuli produced comparable effects to “intermixed” adaptors, which had a more uniform appearance. Also in Experiment 3, remote TAEs were observed when quantization of the orientation gradient resulted in the adapting texture being perceptually segregated into vertical bands (see Figure 5B). It is unlikely therefore, that these effects are a simple by-product of the texture segmentation process.

We have previously hypothesized that remote TAEs could arise via a reciprocal interaction between local orientation coding mechanisms in V1 and extrastriate visual areas tasked with extracting global orientation structure (Roach et al., 2008). This suggestion was motivated by psychophysical studies showing that extraction of global form structure involves the pooling of local orientation signals across space and spatial scale (Dakin and Bex, 2001; Achtman, 2003) and anatomical and physiological studies showing a close alignment of feedforward and feedback connections between V1 and extrastriate areas (e.g., Angelucci et al., 2002). We reasoned that if feedback to V1 acts to inhibit *all* local orientation detectors over which a second-stage unit receives input, any resulting effects ought to show a loss of selectivity commensurate with nature of the feed-forward pooling. According to this idea, adapting to a globally structured stimulus could produce selective suppression of V1 neurons with orientation preferences matching the orientation structure, but where receptive field position and/or spatial frequency tuning dictate that they are relatively unresponsive to the adapting stimulus. This in turn would be sufficient to drive TAEs in regions of space where the adapting pattern was occluded and that are tolerant to changes in spatial frequency (see Roach et al., 2008 for further details). The notion of feedback suppressing activity in V1 that is consistent with orientation structure represented in higher visual areas is broadly suggestive of some form of predictive coding (Mumford, 1991; Rao and Ballard, 1999; Spratling, 2010). However, interpretation of remote TAEs within this framework is not straightforward. One complicating factor is that our paradigm measures changes in orientation perception occurring within a region of space in which the adapting stimulus is occluded. In predictive coding models, feedback functions to remove or reduce activity in lower areas that matches the predictions of higher areas. In the sub-population of V1 neurons representing the occluded region of space however, there may be little or no activity to “explain.” There is some evidence suggesting that feedback continues to contribute to V1 activity in the absence of any feed-forward stimulation, but these effects are not typically accounted for by predictive coding models (see Muckli and Petro, 2013 for a recent review). A second issue is that rather than a modulation of ongoing activity during the presentation of a structured stimulus, explanation of remote TAEs requires a lasting and selective change in neural responsivity. Notionally, visual adaptation can be conceived as predictive coding operating in time. However, formal model implementations of this process are currently lacking.

Why does the visual system adapt to local orientations that are implied by the structure of a texture, but not actually present in the image? One situation in which this could be functionally advantageous is when regions of a scene are temporarily occluded from view. A consequence of having coherent spatial structure in an image is that the composition of occluded areas can be predicted from the surrounding spatial context. Adaptation mechanisms could exploit this predictability to mimic the processing that would have occurred with full viewing of the scene, thereby preparing the visual system for when the occlusion is removed. When a region of the visual field is deprived of input for long period of time (e.g., patients with scotoma), perceptual filling-in of texture and other image properties often occurs (Gerrits and Timmerman, 1969; Ramachandran and Gregory, 1991). Remote TAEs may reflect the operation of a shorter-term neural filling in process, one that changes the adaptive state of local orientation detectors without generating a conscious percept.

### Conflict of interest statement

The authors declare that the research was conducted in the absence of any commercial or financial relationships that could be construed as a potential conflict of interest.
